# Helix 8 of the angiotensin- II type 1A receptor interacts with phosphatidylinositol phosphates and modulates membrane insertion

**DOI:** 10.1038/srep09972

**Published:** 2015-06-30

**Authors:** Daniel J. Hirst, Tzong-Hsien Lee, Leonard K. Pattenden, Walter G. Thomas, Marie-Isabel Aguilar

**Affiliations:** 1 Department of Biochemistry and Molecular Biology, Monash University, Melbourne 3800 Australia; 2 School of Biomedical Sciences, The University of Queensland, St Lucia, Queensland, 4072 Australia

## Abstract

The carboxyl-terminus of the type 1 angiotensin II receptor (AT_1A_) regulates receptor activation/deactivation and the amphipathic Helix 8 within the carboxyl-terminus is a high affinity interaction motif for plasma membrane lipids. We have used dual polarisation interferometry (DPI) to examine the role of phosphatidylinositdes in the specific recognition of Helix 8 in the AT_1A_ receptor. A synthetic peptide corresponding to Leu^305^ to Lys^325^ (Helix 8 AT_1A_) discriminated between PIPs and different charges on lipid membranes. Peptide binding to PtdIns(4)P-containing bilayers caused a dramatic change in the birefringence (a measure of membrane order) of the bilayer. Kinetic modelling showed that PtdIns(4)P is held above the bilayer until the mass of bound peptide reaches a threshold, after which the peptides insert further into the bilayer. This suggests that Helix 8 can respond to the presence of PI(4)P by withdrawing from the bilayer, resulting in a functional conformational change in the receptor.

G protein-coupled receptors (GPCRs) are the largest superfamily of cell surface receptors^1^. As GPCRs are key drug targets^2^, elucidating the mechanisms that activate/deactivate GPCRs is fundamental to expanding our understanding of these receptors and the development of new therapeutics. The signature motif of GPCRs consists of seven transmembrane-spanning helices that form an extracellular/intra-membrane ligand binding pocket and a cytoplasmic face that couples to G proteins and promotes signalling. The angiotensin type 1 receptor (AT1R) is a 359 amino acid GPCR that mediates the important cardiovascular and homeostatic actions of the peptide hormone, angiotensin II (AngII)^3^,^4^. It couples primarily to the heterotrimeric G protein, G_q/11_, to activate phospholipase Cα (PLCα), which hydrolyses phosphatidylinositol (4,5) bisphosphate (PtdIns(4,5)P_2_, PIP2) to generate the soluble second messengers, diacylglycerol (DAG) and inositol (1,4,5) trisphosphate (IP_3_)^5^. These messengers in turn activate protein kinase C and raise intracellular calcium respectively, thereby promoting cellular responses that are the basis of AngII actions (principally, vasoconstriction, aldosterone release, thirst, and salt appetite)^3^. Inappropriate activity of the AT_1_R signalling system leads to hypertension and cardiac, renal and vascular hypertrophy and non-peptide antagonists of the AT_1_R are used to lower blood pressure, alleviate cardiovascular dysfunction and to prolong life.

A highly conserved feature of the prototypic seven-transmembrane spanning arrangement of GPCRs is an additional helix (termed Helix 8), which comprises the first 15–20 amino acids of the intracellular C-terminus and is positioned parallel to the lipid bilayer^3^,^4^,^6^. This region of the GPCR is a focal point for many protein-protein interactions that are crucial for receptor coupling to G proteins and other signalling/regulatory molecules. We and others have previously shown that a peptide corresponding to helix 8 of the AT_1_R (Leu^305^ to Lys^325^, henceforth referred to as AT_1_R-H8) displays high affinity binding to CaM in the presence of calcium^7^,^8^. We have also previously used synthetic peptides and model membranes to show that AT_1_R-H8 binds with high affinity to phospholipid bilayers via both electrostatic and hydrophobic interactions^9^,^10^,^11^. A subset of Helix 8 sequences include one or more cysteines which can be acylated (e.g. by palmitoylation)^10^. Thus, this α-helical region is more likely tethered to the plasma membrane rather than extending into the cytoplasm, and indeed crystal structures of GPCRs, such as the β-adrenergic receptor and the A2A adenosine receptor, indicate that this region does oppose the inner leaflet of the plasma membrane^12^,^13^,^14^. Many GPCRs possess similar, putative helical extensions from TM7, indicating that, in the absence of acylation on cysteine residues, some receptor tails may dynamically attach to the membrane through side chain-lipid interactions to regulate local receptor structure and function.

The role of the lipid components of the plasma membrane is therefore not only to provide a physical barrier to the cell, but to act as dynamic regulators of integral membrane protein structure and function^15^,^16^,^17^ and our studies suggest that AT_1_R-H8 plays a central role in receptor function and that the plasma membrane is an important regulator of that function^10^,^11^,^18^. Anionic phospholipids, including PIPs, interact electrostatically with polybasic proteins^16^,^19^,^20^,^21^, and the Helix 8 region of GPCRs, with its multiple basic residues, is a likely candidate for this interaction. Several studies indicate that anionic phospholipids, including phosphatidylserine (PS) and PIPs, may cluster to form microdomains that are significant for intracellular signalling^18^,^22^,^23^,^24^. In the present study, we use dual polarization interferometry (DPI) to analyze the binding of the binding of AT_1_R-H8 to various bilayer types. The data are analysed using the kinetic methods we introduced specifically for analysing DPI data^25^, which allow for the combined fitting of mass and birefringence data, and also account for the possibility of lateral expansion in the lipid bilayer as a result of peptide-membrane interactions. This new approach therefore not only allows the binding to be analysed, but also allows changes in membrane structure to be unveiled and quantified. We present evidence that the key components of the membrane for this interaction are the phosphatidylinositols and we have investigated a specific interaction between AT_1_R-H8 and the phosphatidylinositol phosphate lipids (PIPs – referring collectively to all species).

## Results

### Deposition of lipid bilayers

DMPC is a zwitterionic bilayer type with overall neutral charge and represents a control for other bilayers with other properties. Since the main aim is to investigate the specific binding of Helix 8 to PIPs, it is important to also control for the possibility that any changes in binding result from of an overall increase in charge rather than specific binding to PIPs, as such non-specific changes in binding have been previously observed^25^. The additional components of the phospholipid mixture were selected to mimic the intracellular leaflet of the plasma membrane where Helix 8 is located. DMPS is present in the inner leaflet of the eukaryotic plasma membrane, so its inclusion in the bilayers allows a more accurate representation of biological conditions. PI(4,5)P_2_ was chosen as it is well known as a lipid signalling molecule, and is one of the downstream effectors of AT_1_R signalling. PI(4)P was chosen for comparison, to investigate whether the quantity and positioning of phosphate groups is significant. Each bilayer was deposited according to methods previously described^25^,^26^,^27^,^28^ and the properties of each lipid bilayer are listed in Table 1 and reveal that the thickness, mass and birefringence values were highly consistent.

### AT_1_R-H8 Membrane Binding Characteristics

#### Mass and birefringence versus time

**Figure 1:**
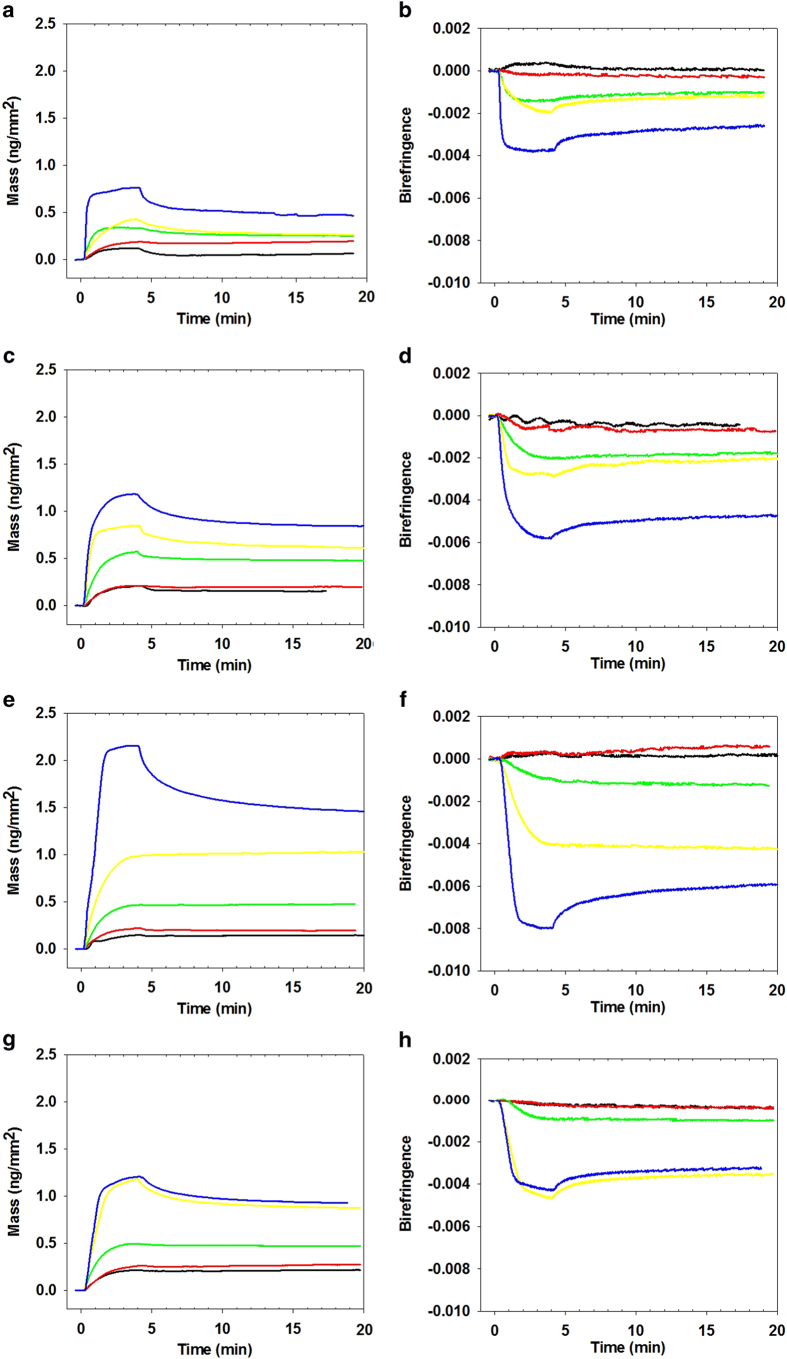
Plots of changes in mass and birefringence versus time for AT_1_R-H8 at concentrations 0.5 μM (black line), 1 μM (red line), 2 μM (green line), 5 μM (yellow line), and 10 μM (blue line): binding to lipids DMPC (mass changes vs. time, (**A**); birefringence changes vs. time, (**B**); DMPC/DMPS (mass changes vs. time, (**C**); birefringence changes vs. time, (**D**); DMPC/DMPS/PI(4) P (mass changes vs. time, (**E**); birefringence changes vs. time, (**F**); DMPC/DMPS/PI(4,5)P_2_ (mass changes vs. time, (**G**); birefringence changes vs. time, (**H**).

The changes in membrane order as a result of AT_1_R-H8 binding were quantitatively examined through the measurement of the changes in optical birefringence (∆*n*_*f*_) of the bilayer simultaneously with the membrane-bound peptide mass (*m*_*p*_) in real time as previously described^26^,^27^,^28^,^29^,^30^. In particular, the effect of lipid composition on the binding of AT_1_R-H8 to the membrane mimics (DMPC, DMPC/DMPS (80:20), DMPC/DMPS/PI(4)P(76:20:4) and DMPC/DMPS/PI(4,5)P_2_ (76:20:4)) was analysed by DPI. Peptide solutions of increasing concentration (0.5–10 μM) were introduced to freshly prepared bilayers. Fig. 1 shows the plots of mass changes (A, C, E, G) and birefringence changes (B. D, F, H) versus time. The plots of mass vs. time show an initial binding phase (with peptide solution flowing over the bilayer and binding to the membrane) followed by a dissociation phase (with bulk buffer flowing over the bilayer) where the peptide dissociates from the membrane (here we use “mass change” and “birefringence change” to refer to the difference between the measured mass or birefringence and that of the fresh lipid bilayer). Mass changes increased during the binding phase as the peptide bound to the bilayer, and for higher concentrations some, but not all, of the bound peptide dissociated from the bilayer during the dissociation phase. Very little peptide dissociation was observed for lower concentrations. Overall AT_1_R-H8 binding to the anionic membranes was higher than to the zwitterionic DMPC, with even higher binding to DMPC/DMPS/PI(4)P, both in terms of mass change and birefringence change. For the plots of birefringence changes with time (Fig. 1b, d, f and h), a similar trend to the mass changes was observed but with a negative sign, with birefringence decreasing in the binding phase, but recovering during the dissociation phase for higher concentrations.

#### Trends in mass and birefringence changes vs. concentration

**Figure 2:**
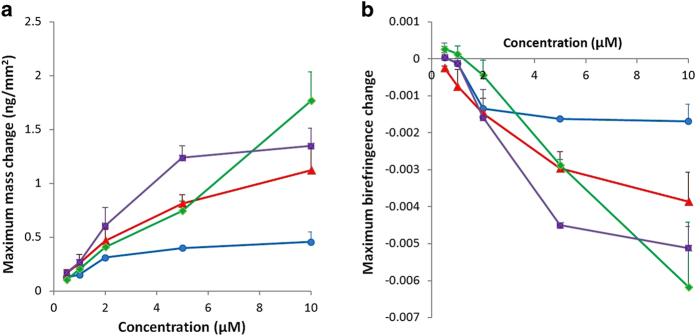
Maximum mass change (vertical axis, Fig. 2a) and birefringence change (vertical axis, Fig. 2b) for AT_1_R-H8 binding to DMPC (blue circles), DMPC/DMPS (80:20) (red triangles), DMPC/DMPS/PI(4)P (76:20:4) (green diamonds), and DMPC/DMPS/PI(4,5)P_2_ (76:20:4) (purple squares), for peptide concentrations ranging from 0.5 to 10 μM (horizontal axis). Error bars show one standard error from at least 3 independent experiments. The maximum birefringence change is defined as the greatest deviation (whether positive or negative) during the injection period relative to the baseline before the injection.

The maximum mass change for each concentration and lipid type is shown in Fig. 2a. AT_1_R-H8 binding to DMPC was relatively low, at about 0.3 ng/mm^2^ at 2 μM concentration, and increased only slowly with higher concentrations to about 0.45 ng/mm^2^ at 10 μM concentration. Binding to DMPC/DMPS was significantly higher, reaching 0.45 ng/mm^2^ at 2 μM and 1.1 ng/mm^2^ at 20 μM. This increase is likely due to the negative charge of the DMPS molecules more strongly attracting the positively charged residues of AT_1_R-H8. For concentrations up to 5 μM the mass of peptide bound to DMPC/DMPS/PI(4)P was very similar to that for DMPC/DMPS; however, for the highest concentration (10 μM), the amount of binding to the PI(4)P-containing membrane continued to increase markedly, almost doubling from 5 μM to 10 μM, whereas for DMPC/DMPS there was only a slight increase in binding. For DMPC/DMPS/PI(4,5)P_2_ a different pattern was seen, where the binding exceeded that for DMPC/DMPS and DMPC/DMPS/PI(4)P at 2 and especially 5 μM, but did not increase further at 10 μM.

The birefringence changes for each lipid type at various concentrations of AT_1_R-H8 are shown in Fig. 2b. At higher concentrations, the trends for birefringence mirror the trends for mass, with higher mass levels corresponding to more negative birefringence changes. However, at lower concentrations, DMPC/DMPS/PI(4)P undergoes a *positive* birefringence change at the lowest concentrations, 0.5 and 1 μM, and only a very small negative birefringence change (compared, for example, to DMPC/DMPS which has similar mass binding) at 2 μM. A similar, but less marked, effect can be seen for DMPC and DMPC/DMPS/PI(4,5)P_2_ which exhibited almost no birefringence change at 0.5 and 1 μM, compared to DMPC/DMPS which had a fairly consistent ratio between mass and birefringence changes regardless of concentration.

### AT_1_R-H8 Induced Changes in Membrane Order

#### Qualitative interpretation of birefringence-mass results

**Figure 3:**
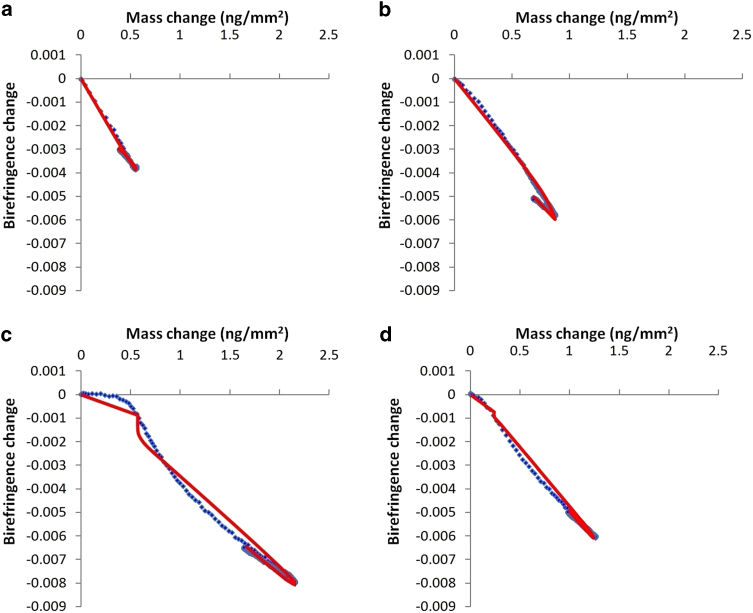
Plots of birefringence vs. mass for the experimental data and a selected model for AT_1_R-H8 binding to various lipid bilayers. The model selected gives the best balance between a good fit and avoiding excessive parameters that only marginally improve the fit. (**A**): experimental data for Helix 8 binding to DMPC (blue dots), with the three-state model with bilayer expansion fitted to the data (red line). (**B**): experimental data for Helix 8 binding to DMPC/DMPS (blue line), with the two-state model fitted to the data (red line). (**C**): experimental data for Helix 8 binding to DMPC/DMPS/PI(4)P (blue line), with the three-state model with bilayer expansion and mass threshold fitted to the data (red line). (**D**): experimental data for Helix 8 binding to DMPC/DMPS/PI(4,5)P_2_ (blue line), with the three-state model with bilayer expansion and mass threshold fitted to the data (red line).

Analysis of the changes in the membrane structure upon peptide binding reveals a possible mechanism of AT_1_R-H8 binding in terms of surface association and membrane structural changes. For this analysis, the birefringence-mass plots for 10 μM AT_1_R-H8 show the qualitative features of AT_1_R-H8 binding and will be quantitatively analysed in the following section. Plots of mass and birefringence vs. time are shown in Figs 1,2, respectively. For DMPC (Fig. 3a), the relationship between birefringence and mass is linear, with mass increasing and birefringence decreasing during the binding phase, and mass decreasing and birefringence increasing during the dissociation phase. The amount of binding on DMPC was less than for other bilayer types. For DMPC/DMPS (Fig. 3b) the plot is similar, but somewhat less linear, with a shallower gradient during the initial binding, and the graph did not exactly retrace the binding phase during the dissociation phase (the birefringence is slightly lower). However, for DMPC/DMPS/PI(4)P (Fig. 3c), the pattern was markedly different; the graph traced horizontally (with little change in birefringence) until the mass reached about 0.4 ng/mm^2^, at which point the birefringence dropped rapidly until reaching approximately the same birefringence-mass change ratio as for DMPC/DMPS. The dissociation phase retraced the final stages of the binding phase almost exactly. DMPC/DMPS/PI(4,5)P_2_ (Fig. 3d) showed a moderately curved pattern more similar to that for DMPC/DMPS, but with the dissociation phase retracing the binding phase more closely.

#### Kinetic modelling of AT_1_R-H8-membrane interactions

**Figure 4:**
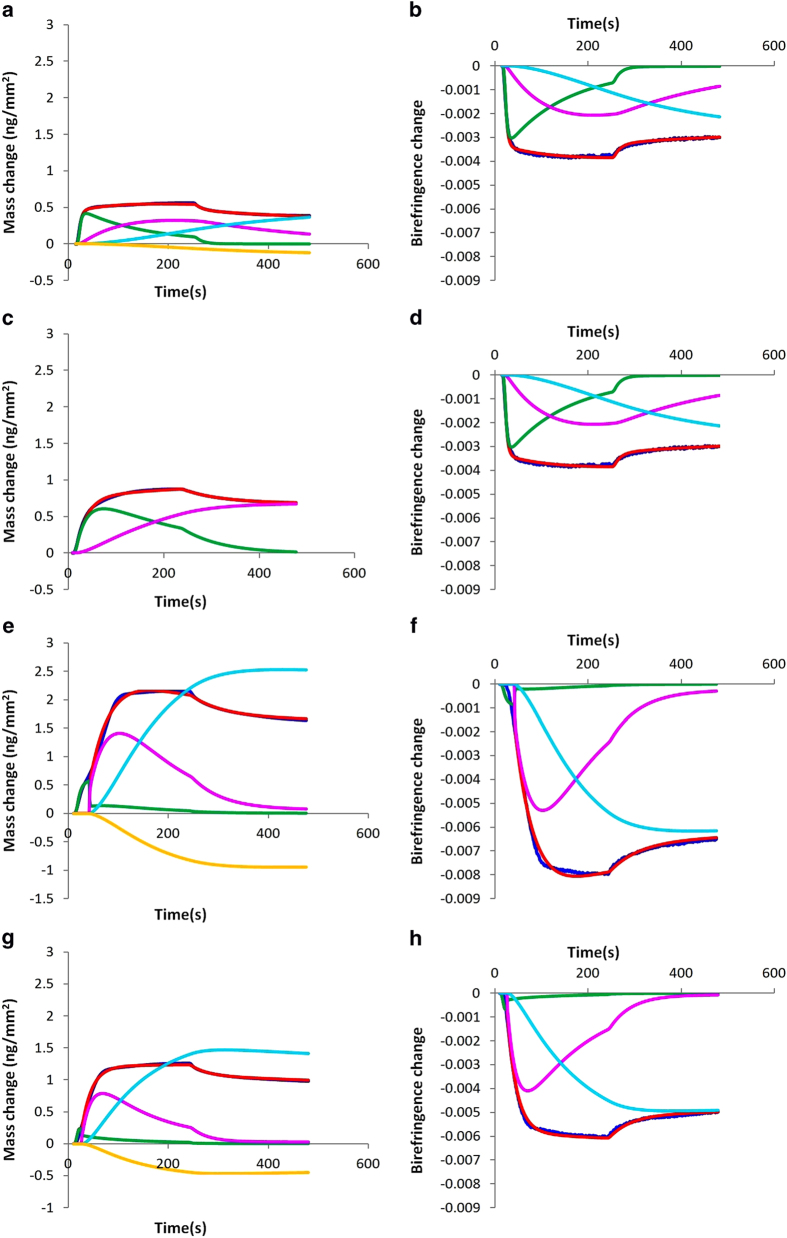
Deconvolution of peptide states for mass and birefringence change vs. time for the binding of 10 μM Helix 8 for lipid bilayers and model corresponding to Fig. 3, showing, where applicable, experimental data (blue line) and the model mass/birefringence change (red line), first state (green line), second state (magenta line), third state (cyan line), and effect of bilayer expansion (yellow line). Graphs are for the following lipids and models: DMPC using a three-state model with bilayer expansion, mass changes (**A**) and birefringence changes (**B**); DMPC/DMPS using a two-state model without bilayer expansion, mass changes (**C**) and birefringence changes (**D**); DMPC/DMPS/PI(4)P using a three-state model with a mass threshold and bilayer expansion, mass changes (**E**) and birefringence changes (**F**); DMPC/DMPS/PI(4,5)P_2_ using a three-state model with a mass threshold and bilayer expansion, mass changes (**G**) and birefringence changes (**H**).

Kinetic modelling of the mass and birefringence data was performed for each binding event using a set of related models. The normalised least-squares errors for the best fit for each model on each bilayer are listed in Table 2. The *two-state model* was the simplest model used, and represents the process where a peptide binds to the surface, forming the first state as a combination of peptide and associated lipids as previously described^25^. This first state then proceeds to either dissociate from the surface or convert into the second state, which may then return to the first state. The graphical output of each fit is shown in Fig. 4 and the rate of each change is represented by a kinetic constant as listed in Table 3. Additionally, each of the two membrane-bound states is assumed to affect the birefringence of the lipid bilayer proportionally to the mass of peptide bound in that state; the relative magnitude of this effect is described by the constants n_1_ and n_2_ (Table 3). The *three-state model* is a logical extension of the *two-state model* where the second membrane-bound state may convert to a third state, and vice versa. In addition, these models often need to be modified to take into account *bilayer expansion*, where the mass per unit area of the lipid bilayer decreases as a result of peptide state conversions. In the cases presented here, it is assumed that only the last state (the second state of the two-state model, and the third state of the three-state model) results in bilayer expansion.

Other new model features were developed to account for the unusual binding process seen with DMPC/DMPS/PI(4)P, where the loss in birefringence was very small at first and then dropped rapidly later in the binding phase. Two hypotheses were suggested: either the bilayer responds very slowly to the binding of peptide resulting in a substantial delay between binding and birefringence changes (resulting in the *birefringence-lag model*); or the conversion from the first state to second state is prevented until a certain threshold for mass of peptide per unit area is passed (resulting in the *mass-threshold model*). It is acknowledged that, given the complexities of lipid-protein interaction, these models would only represent an approximation of the physical processes occurring, and should not be expected to provide an exact fit to the data.

The two-state model gave a reasonable fit to the DMPC binding curve (Fig. 4a,b) with a combined normalized least-squares error (hereafter referred to merely as the *error*) of 0.389, reduced slightly to 0.356 taking into account bilayer expansion. The three-state fit reduced the error to 0.177, about half the error of the two-state fit. However, the change from two-state to three-state also adds three parameters to the model, so whether the three-state fit makes a significant improvement here is not clear. The more complex birefringence lag model and mass threshold model did not make a noticeable improvement to the fit.

The two-state model gave a very close fit to the DMPC/DMPS binding curve (Fig. 4c,d), with an error of just 0.105, which was not significantly further improved by including bilayer expansion, birefringence lag or a mass threshold. The three-state model improved the error further to 0.085, and the addition of bilayer expansion further reduces this to 0.051. Similar to DMPC, this represents about half the error for the two-state model, and so once again it is difficult to conclude whether this represents a real difference in mechanism, especially given the original two-state model gave a very good fit.

The two-state model and three-state model gave very poor fits to the DMPC/DMPS/PI(4)P binding curve (Fig. 4e,f), both with and without bilayer expansion. However, the three-state model gave a very significant improvement in fit with the use of birefringence lag or mass threshold, with the error dropping from 0.796 for the three-state model with bilayer expansion to 0.319 (with birefringence lag) or 0.303 (for the mass threshold model) respectively. For the mass-threshold model, there was a very small n_1_ value, indicating little disruption of the bilayer in the first state, while the n_2_ and n_3_ values were more comparable to those observed with other lipid bilayer types.

For comparison, kinetic modelling was also used to fit the 5 μM concentration of AT_1_R-H8 on DMPC/DMPS/PI(4)P (Figure S1, Table S1). A two-state model was sufficient to give a very close fit; similarly to the 10 μM concentration, the first state had a very small birefringence-mass coefficient n_1_, and the second state was once again much larger. However, there was no need for a third state, or a mass threshold. This suggests that the first two states are characteristic for this peptide/lipid combination, whereas the dynamics giving rise to the appearance of a third state may only be apparent at higher concentrations. It is notable that the 10 μM concentration resulted in significant reduction in mass (presumably from peptide dissociation) in the dissociation phase, whereas there was almost no dissociation for the 5 μM concentration (a pattern consistent across multiple experiments).

The result of kinetic modelling for the DMPC/DMPS/PI(4,5)P_2_ is somewhat intermediate between that for DMPC/DMPS and DMPC/DMPS/PI(4)P (Fig. 4g,h). The two-state model has an error of 0.837, which improves to 0.422 with bilayer expansion. The three-state model, even with bilayer expansion, only improves this slightly, to 0.369, however once birefringence lag is included the gap between two-state and three-state models widens, with errors of 0.367 and 0.153 respectively. The use of the mass threshold also decreases the error to 0.169 for the three-state model.

The kinetic model can further be used to split, or *deconvolute*, the mass and birefringence signals by each proposed state, and in the case of mass, the bilayer expansion effect (where the expansion of the bilayer as a result of peptide binding causes a reduction in mass), as shown in Fig. 4 for the selected models. Note the similarity of the DMPC/DMPS/PI(4)P and DMPC/DMPS/PI(4,5)P_2_ states, suggesting that the two may follow similar mechanisms in binding even though the birefringence-mass plots appear superficially different. These cannot be compared to the DMPC/DMPS model as any three-state model for the DMPC/DMPS binding is degenerate (there are many different equally good fits).

## Discussion

The concept of a helical domain interacting with PIPs (with PIP2 being the substrate of G_q/11_-PLC IP3 generation) is novel for GPCRs but is an emerging theme for a number of membrane receptors and ion channels^22^,^24^,^31^. However a detailed biophysical characterisation of these interactions is lacking. In this study we used DPI to analyze the binding of the binding of AT_1_R-H8 to various bilayer types, some of which include PIPs. DPI is an optical biosensor technology which not only provides information on binding interactions but also allows the measurement of changes in membrane structure in terms of thickness and bilayer order^26^,^27^,^28^,^29^,^32^. We have also previously demonstrated that changes in membrane structure by antimicrobial peptides observed with DPI correlate well with solid state NMR^33^, and also neutron reflectometry^34^. While there has been one previous paper that has studied PIP-containing membranes^35^, our study is the first to use DPI to analyse the very subtle but significant effects of PI(4)P and PI(4,5)P_2_ on the behaviour of a peptide derived from a GPCR. We have also previously demonstrated that changes in membrane structure by antimicrobial peptides observed with DPI correlate well with solid state NMR^33^, and also neutron reflectometry^34^. In the present study we have used mass and birefringence (corresponding to molecular order) to characterise the state of the bilayer, and the results were analysed with multiple-state kinetic modelling techniques to elucidate the intermediate states of peptide binding.

We previously used two-state and three-state models with bilayer expansion in our analysis of the antimicrobial peptide HPA3, which were sufficient to approximately fit the main features of its binding to saturated and unsaturated bilayers^25^. However, in the case of the non-cytolytic AT_1_R-H8 binding to DMPC/DMPS/PI(4)P, these models were clearly inadequate, giving large error values. In addition, the fits obtained for the 3-state model were of doubtful plausibility, with large positive n_3_ values (birefringence coefficients for the 3rd state) that seem unlikely to be an accurate reflection of the physical process. Addition of birefringence lag or a mass threshold both improved the fit significantly. The birefringence-lag model suggests a physical mechanism whereby the membrane reacts slowly to the binding of peptide molecules, whereas the mass threshold suggests an immediate change following the mass of peptide on the surface reaching a certain threshold. The mass threshold provided a slightly better fit than birefringence lag for the same number of parameters, and also produced more plausible parameter values. All the birefringence coefficients n_1_, n_2_, n_3_ were small negative numbers when using the mass threshold model, which suggests disruption of the membrane, compared with larger values, including a large positive n_3_ value, for the birefringence-lag model. Thus, it is reasonable to conclude that the mass-threshold model is the best approximation of the actual physical process. We believe the three-state mass-threshold model with bilayer expansion in the third state is the most parsimonious kinetic representation of our data for the binding of Helix 8 to DMPC/DMPS/PI(4)P.

For DMPC/DMPS/PI(4,5)P_2_, the three-state model as used in our previous studies provided an adequate fit, but addition of birefringence lag and a mass threshold further improved the fit. In this case, the birefringence-lag model was a slightly closer fit, but once again the mass-threshold model provided more plausible parameter values. Although the effect is not as dramatic as for DMPC/DMPS/PI(4)P, the newly developed models provide a significant improvement in fit, with less than half the error for the addition of one parameter. This improvement was not observed for the two-state model with a mass threshold, and only a small improvement was seen using the two-state model with birefringence lag.

**Figure 5:**
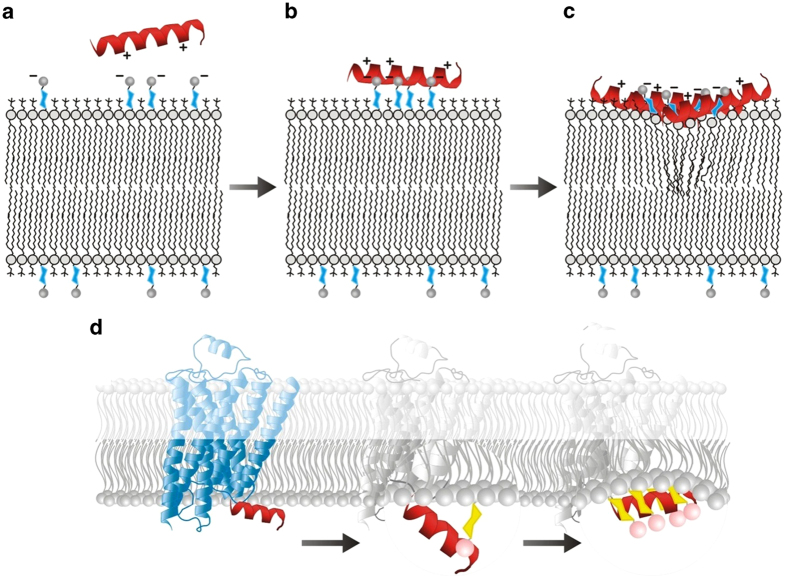
Schematic for the proposed mechanism for the binding of Helix 8 to a lipid bilayer containing PIPs, as observed by DPI. Before binding (**A**) the positive residues of the Helix 8 peptide are attracted to the negatively charged phosphate groups on the PIPs. Upon binding (**B**) the helix is held above the membrane by ionic bonding between Helix 8 and PIP phospholipids. As a large amount of Helix 8 peptide binds to the membrane (**C**) the ratio of PIPs to peptide drops and the peptide moves into the membrane, causing disruption of its structure. (**D**) Schematic for the proposed mechanism by which interaction between Helix 8 and PIPs may trigger a conformational change within the context of the AT_1_R. The first change involves ionic binding to the phosphate groups of PIP molecules protruding from the membrane surface, pulling the helix away from the membrane. This results in the unfavourable exposure of hydrophobic residues to the hydrophilic cytosol. In the second change, the helix responds to this by returning to the inner leaflet of the membrane, lying between the phosphate groups of the PIPs and the headgroups of the more abundant lipid species.

Based on the results of these models, we conclude that the two-state model, even without bilayer expansion, is sufficient to represent the simpler DMPC and DMPC/DMPS bilayers. However, for membranes containing PIPs, the mass threshold improved the model fit substantially when used with the three-state model with bilayer expansion, and the effect was especially marked for DMPC/DMPS/PI(4)P, but also to a lesser extent for DMPC/DMPS/PI(4,5)P_2_. This leads to the question: what physical processes do these models represent ? A notable feature of both cases (more prominently for DMPC/DMPS/PI(4)P) is that the first birefringence coefficient (n_1_) is significantly lower than the second (n_2_) - this is not surprising given the birefringence-mass plots both begin with a shallower gradient. This means that the initial stage of binding has a relatively small effect on membrane order. The small initial effect of bilayer order suggests that the peptide is held above the interior of the membrane by the strong phosphate charges of the PIP head-groups (shown schematically in Fig. 5a,b), and that it only penetrates further into the membrane when a critical mass of peptide has accumulated on the surface (approximately represented by the mass threshold parameter *T*_m_ (Fig. 5c)). This is further supported by the results for birefringence changes at lower concentrations (≤1 μM, when the critical mass of peptide is never reached) for which there was negligible, or even positive, birefringence change, and significant drops in birefringence were only seen at higher concentrations. If the presence of PIPs holds the peptide above the surface in this way, then in the context of a full GPCR this would allow AT_1_R-H8 to act as a sensor for the presence of PIPs in the membrane, based on whether it sinks into the membrane (in the absence of PIPs) or is held above it (in their presence) as shown in Fig. 5d. The change in position of Helix 8 relative to the membrane could then trigger a conformational change in protein structure, resulting in modulation of function as a result of Helix 8 encountering PIPs in the membrane. A possible structural explanation for the considerable difference between binding to PI(4)P and PI(4,5)P_2_ is that in the sugar ring the 4-phosphate is directly opposite the “tail” of the PIP molecule, and probably more distant from the bilayer than the 5-phosphate - so for PI(4)P the region of negative charge is centered further away from the bilayer compared to PI(4,5)P_2_, strengthening the threshold effect. Significantly, the observations of the striking difference between mechanism of binding to DMPC/DMPS and to DMPC/DMPS/PI(4)P is only possible due to the ability of DPI to measure changes in the membrane structure. In the absence of the birefringence data, mass-only measurements (e.g. with SPR), only reveal increased binding to PI(4)P, and the effect of PIPs on membrane structure changes that control Helix 8 orientation are invisible.

Previous research had indicated that Helix 8 of the angiotensin receptor, and other GPCRs, binds to and interacts with the inner leaflet of the plasma membrane^9^,^10^,^11^. In this study, we have demonstrated a dramatic change in the nature of this interaction following the addition of PIP4 - but not other PIPs - to the membrane, suggesting that AT_1_R-H8 plays a role in detecting changes in the composition of the plasma membrane. This observation was only possible due to the ability of DPI to detect changes in birefringence, representing the molecular order of the bilayer, as changes of mass bound to the bilayer are not clearly different between different bilayer types. In conclusion, we propose a hypothesis for the interaction between Helix 8 and PIP4, whereby the PIP4 molecules pull the AT_1_R-H8 peptide outward from the bilayer, preventing insertion and bilayer disruption until the amount of AT_1_R-H8 peptide on the surface reaches a critical density, at which point it re-inserts. This hypothesis is supported by kinetic modelling of the mass and birefringence changes, using a modified version of our original kinetic modelling technique for the DPI^25^. This biophysical study of the binding mechanism has therefore revealed the influence of lipids on AT_1_R-H8 membrane binding and insertion, and provided new insight into the process by which bilayer signals are converted to changes in Helix 8 and hence possible conformational changes in the protein.

## Materials and Methods

### Chemicals and reagents

1,2-Dimyristoyl-sn-glycero-3-phosphocholine (DMPC), 1,2-dimyristoyl-sn-glycero-3-phospho-L-serine (sodium salt) (DMPS), L-α-phosphatidylinositol-4-phosphate (Brain, Porcine) (ammonium Salt) (PI(4)P) and L-α-phosphatidylinositol-4,5-bisphosphate (Brain, Porcine) (ammonium salt) (PI(4,5)P_2_) were of analytical grade and were purchased from Avanti Polar Lipids (Alabaster, AL, USA). 4-Morpholinepropanesulfonic acid (MOPS), sodium dodecyl sulphate (SDS), calcium chloride, and sodium chloride, all analytical grade, were purchased from Sigma–Aldrich (St Louis, MI, USA). Chloroform, methanol, and ethanol, all HPLC-grade, were purchased from Merck (Darmstadt, Germany). Hellmanex II was purchased from Hellma (Müllheim, Germany). Water was quartz-distilled and deionised using a Milli-Qsystem equipped with UV oxidation to remove organic residues. (Millipore, Bedford, MA, USA). AT_1_R-H8 (LGKKFKKYFLQLLKYIPPKAK) was purchased from GL Biochem (Shanghai, China), and purified to >95% purity by reversed phase HPLC and analysed by LC-MS.

### Preparation of liposomes

Stock solutions of 2 mM DMPC, DMPS (in 3:1 chloroform–methanol), PI(4)P (in CHCl3/MeOH/H2O (20:9:1)), and PI(4,5)P_2_ (in CHCl3/MeOH/H2O (20:9:1)), were prepared; after setting aside sufficient DMPC and POPC these were then mixed to form DMPC–DMPS (molar ratio 80:20), DMPC–DMPS–PI(4)P (molar ratio 76:20:4) and DMPC–DMPS–PI(4,5)P_2_ (molar ratio 76:20:4) solutions. Aliquots containing 0.8 μmol of each lipid mixture used (DMPC, DMPC–DMPG, POPC, POPC–POPG) were dried by use of a gentle stream of N2 gas in a Pyrex test tube, and vacuum dried overnight to form lipid films. These were then hydrated with 10 mM MOPS buffer, 150 mM NaCl, pH 7 buffer at 37 °C in a shaker–incubator for 1 h; the samples were then ultrasonicated in a bath-type sonicator for 30 min, generally resulting in a clear solution. This solution was extruded 19 times through a 100 nm polycarbonate membrane, by means of an Avestin Liposofast extruder (Avestin, ON, Canada).

### Dual polarization interferometry

The details of the technique, including the calculation of mass and birefringence from raw data, were described in our previous reports^26^,^27^,^36^. Briefly, the buffer (10 mM MOPS, 150 mM NaCl, pH 7) and unmodified silicon oxynitride chip were calibrated as previously described^36^. The unilamellar DMPC and POPC bilayers were prepared by depositing the 100 nm liposome solution on the chip in the presence of 1 mM CaCl_2_. The resulting bilayer properties were highly consistent. As the chip was cleaned *in situ* a contact angle measurement could not be directly measured; however, a previous study found contact angles as low as 5° after intensive cleaning *ex situ*^37^; a less severe cleaning procedure produced a contact angle of 32.1°^38^, while the expected contact angle for an unmodified chip is 78.17 ± 0.13°^38^. After cleaning the chip surface, the liposome solution (DMPC, DMPC–DMPG, DMPC–DMPG–PI(4)P or DMPC–DMPG–PI(4,5)P_2_) was injected to form a supported planar lipid bilayer. Single injections of AT_1_R-H8 peptide were then made at concentrations of 0.5 μM, 1 μM, 2 μM, 5 μM and 10 μM, each time cleaning the surface and creating a new lipid bilayer. The injections were performed at a flow rate of 40 μL/min of 240 s duration, and were followed by washing with buffer to remove loosely bound peptide. This procedure results in a trace showing binding phase lasting about 200 seconds (once the delay in starting the injection is taken into account), during which the lipid bilayer is exposed to the peptide solution, followed by a dissociation phase lasting for a duration of at least 15 minutes afterwards, during which the bilayer is washed with buffer. Analysis of the raw experimental data using Analight® Explorer gives simultaneous measurements of mass per unit area, and birefringence.

### Calculation of Birefringence

As outlined in several previous studies^26^,^27^,^33^,^36^,^39^,^40^,^41^,^42^,^43^,^44^, the underlying assumptions for DPI analysis of lipid bilayers have been well documented. These studies have shown that determination of both the RI and thickness of the adsorbed film in real time by DPI is valid for a homogenous isotropic adsorbed film. However, this condition is not fulfilled for biomolecular assemblies with highly anisotropic polarizability such as lipid bilayers. The dominating contribution to the optical response for a supported lipid bilayer (SLB) is from the difference in the molecular polarizability of the linearly polarized optical waveguide modes TM and TE. These differences in the polarizability of the two modes lead to three unknown parameters that need to be determined for the SLBs, i.e., thickness, refractive index and birefringence (the difference between the effective refractive indices of the two principal axes, or optical anisotropy). However, only two parameters can be determined from two orthogonal polarizations in DPI. To obtain two parameters for three unknown parameters, one of the three parameters is fixed (assumed to be constant). Thus, either RI or thickness is fixed to calculate the birefringence. In this and other studies^26^,^27^,^33^,^36^,^39^,^40^,^41^,^42^,^43^,^44^ examining the effect of the molecules on the structural organisation of lipid bilayers adsorbed on a planar solid support, changes in birefringence, thickness and hence mass of the layer were determined by assuming a fixed RI of 1.47 for the bilayer. The birefringence and mass of the bilayer can also be calculated by assuming a fixed thickness of 4.7 as used in other works^35^,^39^,^45^. However, both theoretical and experimental validations of this assumption has shown that invalid mass and thickness values are derived if an isotopic adlayer model is used (without taking the anisotropy into account) 39,42,46.

### Kinetic modelling

Kinetic analysis of binding data often involves calculating the equilibrium dissociation constant K_d_, which is based on a number of assumptions; firstly, the values obtained must be at equilibrium, which is not the case for these measurements; secondly, the concept of a single dissociation constant assumes a simple one-state binding model, involving exponential decay in the amount of peptide bound during the dissociation phase. The kinetic data we have obtained do not feature this pattern; instead, during the dissociation phase, after an initial rapid drop (in some cases) the remaining peptide remains firmly bound to the bilayer, thus contradicting the one-state binding model. As a result, we believe the calculation of the dissociation constant and other simple equilibrium-related constants would be misleading for the binding of AT_1_R-H8 to the lipid bilayer, and more sophisticated analysis is required to determine the kinetics of this process.

The modelling techniques used in this study are built on those described in our previous study, in particular the two-state and three-state models including bilayer expansion^25^. Two particular extensions of the three-state model are used here, which we call the *birefringence-lag model* and the *mass threshold model*.

The birefringence-lag model assumes that the birefringence changes resulting from the first state are delayed, this is approximated by allowing the birefringence changes from state 1 at a time *t* to be proportional to the average of the mass over the time period of length 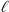, thereby giving the following formula for birefringence *b (t)* at time *t*: 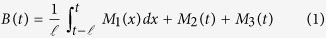
where in this study the integral is evaluated from the data points according to the trapezoidal rule. The birefringence-lag model has one parameter (the time length 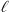) more than the standard three-state model with bilayer expansion.

The mass threshold model involves the introduction of a change in the kinetics of the 1st state → 2nd state transition, such that the rate 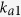 is no longer constant, but instead undergoes a step change when the mass of peptide exceeds a certain threshold value *t*_*m*_. A specific case where 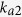 is equal to 0 below the mass threshold may be called the *absolute mass threshold model*, and only requires one more parameter (the threshold value *t*_*m*_) while the more general case also requires as a parameter the association constant that applies below the threshold, which we call 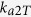. So in the general case the differential equations of the three-state model become:
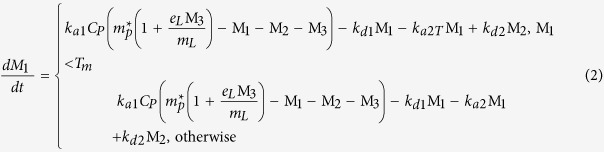

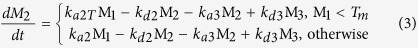

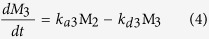
noting that (4) is unchanged from the original three-state model, while (2) and (3) are only split into two cases to take into account the different association constants depending on whether M_1_ is greater than or less than T_m_.

For the mathematical representation of the initial binding of stage 1, we have investigated the effect of substituting the Langmuir stage 1 binding (as presented and used here) with the RSA-based binding, as previously reported^47^ and reviewed^48^; however, that formula for coverage does not produce a superior fit.

#### Physical interpretation of parameters:

***m***_p_*: maximum mass of peptide on membrane assuming full coverage (units of ng mm^−2^).

**k**_**a1**_: binding association constant (units of M^−1^s^−1^).

**k**_**d1**_: binding dissociation constant (units of s^−1^). Proportion of peptides in state 1 that dissociate per second.

**k**_**a2**_: second association constant (units of s^−1^). Proportion of peptides in state 1 that convert to state 2 per second.

**k**_**d2**_: second dissociation constant (units of s^−1^). Proportion of peptides in state 2 that convert to state 1 per second.

**k**_**a3**_: third association constant (units of s^−1^), three-state model only. Proportion of peptides in state 2 that convert to state 3 per second.

**k**_**d3**_: third dissociation constant (units of s^−1^), three-state model only. Proportion of peptides in state 3 that convert to state 2 per second.

**n**_**1**_: *membrane disordering parameter* for state 1 (units of mm^2^ng^−1^). Amount that birefringence changes for each unit of peptide bound in state 1.

**n**_**2**_: *membrane disordering parameter* for state 2 (units of mm^2^ng^−1^). Amount that birefringence changes for each unit of peptide bound in state 2.

**n**_**3**_: *membrane disordering parameter* for state 3 (units of mm^2^ng^−1^), three-state model only. Amount that birefringence changes for each unit of peptide bound in state 2.

**e**_**L**_: Bilayer expansion coefficient (dimensionless). Lipid mass change per unit of peptide in the final state (usually negative, indicating mass loss).

**T**_**m**_: Mass threshold (units of mm^2^ng^−1^). For mass-threshold models: Mass of peptide on the surface that permits the conversion of peptide from state 1 to state 2.


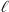
: Birefringence lag (units of s) Average time taken for the lipid bilayer to respond to the binding of the peptide to the bilayer.

**e**_**L**_: Bilayer expansion coefficient (dimensionless). Lipid mass change per unit of peptide in the final state (usually negative, indicating mass loss).

**fit**: Combined least-squares error (see methods) combining mass and birefringence. (lower is better, 0 is a perfect fit, no upper limit)

Parameters kd_1_, kd_2_, kd_3_, ka_2_, and ka_3_ are restricted to a maximum of 0.5 (500 × 10^−3^) to prevent numerical instabilities.

## Additional Information

**How to cite this article**: Hirst, D. J. *et al.* Helix 8 of the angiotensin II type 1a receptor interacts with phosphatidylinositol phosphates and modulates membrane insertion.. *Sci. Rep.* **5**, 9972; doi: 10.1038/srep09972 (2015).

## Figures and Tables

**Table 1:** Properties of supported lipid bilayers as observed by use of dual polarization interferometry techniques at 20 °C.

**Lipid**	**Thickness (nm)**	**Mass (ng/mm2)**	**Birefringence**
DMPC	4.95 ± 0.10	4.98 ± 0.09	0.0206 ± 0.0006
DMPC/DMPS (80:20)	4.79 ± 0.07	4.82 ± 0.07	0.0218 ± 0.0006
DMPC/DMPS/PI(4)P 76:20:4)	4.73 ± 0.21	4.73 ± 0.22	0.0226 ± 0.0009
DMPC/DMPS/PI(4,5)P_2_ 76:20:4)	5.09 ± 0.14	5.12 ± 0.15	0.0229 ± 0.0005

Values are means from 12 observations; the error is given as one standard deviation from the mean.

**Table 2:** Normalized least-squares error for the best fit of model types to the binding curves for AT_1_R-H8 binding to lipid bilayers at 10 μM peptide concentration.

**Lipid composition**	**2-state**	**2-state + expansion**	**2-state + expansion + lag**	**2-state + expansion + threshold**	**3-state**	**3-state + expansion**	**3-state + expansion + lag**	**3-state + expansion + threshold**
DMPC	0.389	0.356	0.356		0.249	0.177	0.177	
DMPC/DMPS (80:20)	0.105	0.104	0.103		0.085	0.056	0.056	
DMPC/DMPS/PI(4)P (76:20:4)	2.168	2.168	1.545	0.919	1.554	0.796	0.319	0.303
DMPC/DMPS/PI(4,5)P_2_ (76:20:4)	0.837	0.422	0.367	0.422	0.756	0.375	0.153	0.169

“Expansion” refers to the use of bilayer expansion in the model, “Lag” refers to the use of the birefringence-lag model; “Threshold” refers to the use of the mass-threshold model.

**Table 3:** Parameters for selected model fits (with selected fits depicted in Fig. 3) for binding of AT_1_R-H8 to lipid bilayers.

**Lipid, Model**	m^*^_p_	**ka**_**1**_	**k**_**d1**_ **x 10**^**−3**^	**k**_**a2**_ **x 10**^**−3**^	**k**_d2_ **x 10**^**−3**^	**k**_**a3**_ **x 10**^**−3**^	**k**_**d3**_ **x 10**^**−3**^	**n**_**1**_ **x 10**^**−3**^	**n**_**2**_ **x 10**^**−3**^	**n**_**3**_ **x 10**^**−3**^	**e**_**L**_	**T**_**m**_	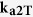 **x 10**^**−3**^	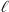	**fit**
**DMPC 2-state**	0.575 (0.559–0.598)	16905 (13473–21741)	24.0 (15.3–37.9)	5.56 (5.14–6.01)	0.20 (0–0.76)			−6.75 (−7.16–6.38)	−7.38 (−7.67–7.12)						0.389
**DMPC** (Fig. 3a)**3-state + bilayer expansion**	0.656 (0.629–0.683)	14682 (12683–17317)	56.6 (48.3–71.8)	10.43 (8.13–11.91)	0.68 (0.33–0.98)	3.29 (1.91–5.37)	0 (0–6.6)	−7.26 (−7.66–6.83)	−6.34 (−6.81–5.82)	−5.83 (−7.44–4.20)	0.34 (0.24–8.65)				0.177
**DMPC/DMPS** (Fig. 3b) **2-state**	0.957 (0.932–0.990)	3416 (3209–3638)	7.0 (6.3–9.6)	5.1 (4.7–5.7)	0 (0–0.38)			−6.05 (−6.24–5.85)	−7.30 (−7.45–7.15)						0.105
**DMPC/DMPS 3-state + bilayer expansion**	1.152 (1.091–1.207)	3063 (2841–3305)	20.6 (17.9–24.5)	15.2 (13.7–16.5)	0 (0–15.1)	5.4 (4.8–6.8)	0 (0–2.4)	−6.27 (−6.52–5.94)	−6.32 (−6.51–6.07)	−4.98 (−5.23 –4.85)	0.35 (0.33–0.40)				0.056
**DMPC/DMPS/PI(4)P** (Fig. 3c) **3-state + expansion + lag**	5.846 (5.220–6.349)	572 (504–600)	0 (0–2.6)	19.5 (17.8–21.4)	1.3 (0–5.4)	13.8 (13.1–14.4)	10.2 (9.3–11.2)	8.17 (6.36–9.71)	−18.54 (–19.71–17.18)	10.11 (8.69–11.71)	0.96 (0.88–1.01)			106.7 (94.9–119.2)	0.319
**DMPC/DMPS/PI(4)P 3-state + expansion + threshold**	3.902 (3.633–4.330)	1847 (1804–1927)	103.7 (8.2–152.7)	500 (273–500)*	33.7 (20.4–69.4)	10.6 (9.1–13.4)	0.38 (0–1.76)	−1.52 (−2.53–0.45)	−3.75 (−4.02–3.51)	−2.43 (−2.51–2.36)	0.37 (0.35–0.39)	0.568 (0.509–0.636)			0.303
**DMPC/DMPS/PI(4,5)P**_**2**_ **3-state + expansion + lag**	6.230 (5.000–7.620)	567 (548–581)	8.4 (5.4–12.2)	44.6 (40.1–50.4)	4.6 (2.1–6.4)	20.6 (18.8–33.1)	32.0 (27.1–40.8)	−10.9 (−12.0–10.0)	3.35 (2.81–3.96)	−8.54 (−9.72–7.48)	1.70 (1.55–1.83)			15.6 (11.3–19.7)	0.153
**DMPC/DMPS/PI(4,5)P**_**2**_ **3-state + expansion + threshold** (Fig. 3d)	2.129 (2.041–2.205)	4736 (4448–4926)	382.4 (229.6–500)*	500 (393–500)*	38.9 (23.9–51.7)	13.9 (11.1–17.4	0.55 (0.11–1.00)	−3.06 (−5.07–0.58)	−4.90 (−5.18–4.65)	−3.31 (−3.39–3.16)	0.33 (0.31–0.35)	0.232 (0.226–0.242)			0.169
